# Regenerative Strategies for Osteoporotic Craniomaxillofacial Bone Defects: Evidence, Translational Gaps, and Future Directions

**DOI:** 10.3390/biomedicines14102204

**Published:** 2026-09-29

**Authors:** Nur Efşan Aydın, Kerem Özden, Alperen Kırkpunar, Sedat Çetiner

**Affiliations:** Department of Oral and Maxillofacial Surgery, Faculty of Dentistry, Gazi University, 06000 Ankara, Turkey; keremozden@gazi.edu.tr (K.Ö.); alperenkirkpunar@gazi.edu.tr (A.K.); scetiner@gazi.edu.tr (S.Ç.)

**Keywords:** osteoporosis, bone regeneration, craniomaxillofacial defects, biological agents, bone tissue engineering

## Abstract

Background: Osteoporosis alters bone remodeling, osteogenic competence, redox and inflammatory homeostasis, and vascular support. These abnormalities may compromise healing of craniomaxillofacial defects, but evidence for regenerative interventions is distributed across mechanistic studies, non-craniofacial osteoporotic models, craniofacial models without osteoporosis, and a smaller body of direct osteoporotic craniofacial evidence. Methods: A literature search was conducted using PubMed and Web of Science. Search terms combined osteoporosis-related terms with bone regeneration/defect healing, craniomaxillofacial anatomy, and intervention-specific terms. No strict publication-date restriction was applied, and studies were selected according to their relevance to the predefined scope. Evidence was interpreted using two complementary dimensions: directness to osteoporotic craniomaxillofacial regeneration and translational maturity. Results: Direct osteoporotic craniofacial preclinical evidence is available for selected interventions, including melatonin and injectable platelet-rich fibrin, whereas vitamin D, teriparatide, bone morphogenetic proteins, mesenchymal stem cell-based therapies, exosomes, hydrogels, nanomaterials, and 3D-printed constructs are supported by different combinations of indirect osteoporotic, non-osteoporotic craniofacial, mechanistic, and clinical evidence. Teriparatide and recombinant human bone morphogenetic protein-2 have comparatively mature clinical pathways, but their clinical evidence is not synonymous with efficacy in osteoporotic craniomaxillofacial defects. Emerging biomaterials can address delivery, retention, oxidative stress, inflammation, angiogenesis, and osteogenic deficits simultaneously, but remain predominantly preclinical. Conclusions: Direct evidence for regeneration of osteoporotic craniomaxillofacial defects remains limited and predominantly preclinical. The most useful translational distinction is whether a strategy has demonstrated defect-level regeneration in an osteoporotic craniomaxillofacial setting, whether it has an established delivery pathway, and whether its added complexity produces a clinically meaningful advantage. Anatomically relevant preclinical evidence should be interpreted as proof of concept rather than as evidence of clinical readiness. Future studies should use clinically matched osteoporotic defect models, report concomitant osteoporosis therapy, and standardize direct regenerative endpoints rather than relying on bone mineral density or mechanistic markers alone.

## 1. Introduction

Osteoporosis is characterized by reduced bone mass, deterioration of microarchitecture, and an imbalance between bone formation and resorption. Its pathophysiology includes increased osteoclast activity, reduced osteoblast survival and differentiation, inflammatory dysregulation, oxidative stress, and age-related changes in osteocyte signaling [1,2,3]. These processes are clinically important in the craniomaxillofacial region because regeneration after extraction, cyst or tumor surgery, trauma, ridge augmentation, sinus augmentation, and implant-related procedures depends on coordinated osteogenesis, vascularization, matrix formation, and remodeling [1].

A central problem in this literature is that improved bone mineral density, trabecular microarchitecture, osteogenic-marker expression, or systemic fracture outcomes are often discussed alongside defect regeneration as though they were equivalent. They are not. A therapy can improve systemic skeletal parameters without having demonstrated new bone formation within an osteoporotic craniofacial defect, and an intervention that performs well in a healthy calvarial model may not retain the same efficacy in an osteoporotic jaw defect [4,5].

Accordingly, osteoporotic craniomaxillofacial regeneration should be approached as a compromised regenerative microenvironment rather than a single deficit in bone formation. This review links osteoporosis-associated barriers to therapeutic targets and then evaluates interventions according to delivery strategy, anatomical context, directness of regenerative evidence, and translational maturity. The contribution of the review is therefore not to catalog individual agents, but to distinguish direct osteoporotic craniomaxillofacial evidence from indirect skeletal, non-osteoporotic craniofacial, mechanistic, and clinical evidence and to identify the most consequential translational gaps [6,7].

## 2. Literature Search Strategy and Evidence Interpretation Framework

A literature search was conducted in PubMed and Web of Science. Search terms were used individually and in combinations across three domains: (1) osteoporosis-related terms (“osteoporosis”, “osteoporotic bone”); (2) regenerative/anatomical terms (“bone regeneration”, “bone healing”, “bone defect”, “craniofacial”, “craniomaxillofacial”, “maxillofacial”, “calvarial”, “mandibular”, “alveolar”); and (3) intervention-specific terms including melatonin, vitamin D, vitamin E, teriparatide, BMP, PRP, PRF, mesenchymal stem cells, exosomes, hydrogels, nanoparticles, smart biomaterials, 3D printing, and bioprinting. Original studies and relevant reviews were considered. Primary studies were prioritized for efficacy-related statements, while reviews were retained mainly for broader mechanistic or contextual synthesis.

Because this is a narrative rather than a systematic review, study selection was based on relevance to the predefined scope rather than a formal systematic-review protocol. Particular emphasis was placed on studies directly evaluating bone-defect healing under osteoporotic conditions and, when available, on craniofacial or maxillofacial sites. Findings from osteoporotic long-bone models, non-osteoporotic craniofacial models, and mechanistic in vitro studies were retained when they clarified biological rationale or translational feasibility, but they were explicitly treated as indirect evidence. For inclusion in Table 1, primary studies were purposively selected to represent the principal intervention categories discussed in this review and to maximize relevance to the review question. Priority was given to studies that met one or more of the following characteristics: (i) use of a verified osteoporotic model; (ii) evaluation of a craniomaxillofacial defect or clinically relevant oral regenerative site; (iii) assessment of direct regenerative endpoints, such as new-bone formation, defect fill, histomorphometry, micro-CT, or structural integration; and (iv) provision of human clinical or translationally relevant evidence. When direct osteoporotic craniomaxillofacial studies were unavailable, anatomically relevant non-osteoporotic craniomaxillofacial studies or osteoporotic non-craniofacial defect studies were included to illustrate the closest available evidence, and their indirectness was explicitly identified. Table 1 is therefore intended as a structured illustrative synthesis rather than an exhaustive inventory of all retrieved studies.

Evidence was interpreted using two descriptive dimensions: evidence directness and translational maturity. Evidence directness was assigned according to the study population/model and anatomical site: (1) human osteoporotic craniomaxillofacial evidence; (2) direct preclinical osteoporotic craniomaxillofacial evidence, defined as an osteoporotic animal model with a craniomaxillofacial bone defect; (3) indirect evidence, comprising either osteoporotic skeletal defect models outside the craniomaxillofacial region or craniomaxillofacial regenerative models without osteoporosis; and (4) mechanistic/in vitro evidence without direct defect-level assessment. Translational maturity was categorized separately using four operational categories based on the stage of application of the specific regenerative intervention for the indication represented. “Established clinical use” was reserved for interventions with regulatory authorization or documented routine clinical use for the corresponding craniomaxillofacial regenerative indication. “Clinically investigated” referred to interventions evaluated in human craniomaxillofacial regenerative studies but not established for the specific indication under discussion. “Preclinical” referred to interventions evaluated for regenerative efficacy in vivo in animal models without corresponding human evidence for that application. “Experimental” referred to approaches supported predominantly by in vitro, mechanistic, or early proof-of-concept evidence without in vivo defect-level regenerative validation. These categories describe stage of translation rather than methodological quality or efficacy. They were used as descriptive tools to clarify the applicability of each study to the review question and were not intended to constitute a validated evidence-grading or formal risk-of-bias system. Accordingly, greater anatomical or biological directness was not interpreted as equivalent to higher methodological quality. Within the craniomaxillofacial category, anatomical directness was additionally considered at the defect-site level. Thus, evidence from a calvarial defect was regarded as direct evidence for osteoporotic cranial defect regeneration, but not as direct evidence for alveolar, peri-implant, sinus, or mandibular regeneration. Evidence was therefore interpreted within its specific anatomical context, and cross-site extrapolation was considered indirect unless supported by studies in the corresponding defect type.

## 3. Osteoporosis-Associated Barriers to Craniomaxillofacial Bone Regeneration

The regenerative phenotype of osteoporosis is produced by interacting abnormalities rather than by low bone density alone. RANK/RANKL/OPG dysregulation favors osteoclastogenesis and accelerates resorption; impaired mesenchymal stem-cell osteogenesis and osteoblast survival reduce matrix deposition; persistent inflammatory signaling impairs osteogenic differentiation; excessive reactive oxygen species damage osteoblast mitochondria and further stimulate resorption; and impaired angiogenic support limits oxygen, nutrient, and progenitor-cell delivery [1,2,3]. These processes create a defect microenvironment in which newly formed tissue may be produced more slowly and remodeled less predictably [3,8].

These abnormalities are especially relevant to craniofacial reconstruction because successful healing requires the integration of graft or scaffold architecture with local vascular supply and mechanical demands. A contained extraction socket, a peri-implant defect, a sinus-augmentation site, a calvarial critical-size defect, and a segmental mandibular defect are not interchangeable regenerative problems [9,10,11]. They differ in geometry, vascularity, criticality, mechanical loading, access, and the amount and type of tissue that must be restored. Thus, evidence from one defect type should not be generalized to another without qualification [12].

For this reason, regenerative strategies should be judged not only by whether they stimulate osteogenic markers but also by whether they address the pathological microenvironment that limits healing, whether they produce measurable defect-level regeneration, and whether their delivery system is compatible with the specific defect. This pathology-target-intervention framework is used throughout the following sections [13,14].

## 4. Bone Grafts and Scaffold-Based Regenerative Approaches

Bone grafting remains central to craniomaxillofacial reconstruction. Autogenous grafts provide osteogenic cells, matrix, and endogenous signaling molecules but are limited by donor-site morbidity and availability. Allografts and xenografts can provide osteoconductive structure, whereas synthetic bone substitute materials such as hydroxyapatite, beta-tricalcium phosphate, biphasic calcium phosphate, and bioactive glass offer reproducible composition and unlimited supply [15,16]. In osteoporotic bone, however, the performance of an osteoconductive material is constrained by the host’s impaired cellular and vascular response [17,18].

Scaffold design therefore cannot be reduced to material identity. Pore size and interconnectivity influence cell infiltration and vascular invasion; degradation must be coordinated with new tissue formation; stiffness and mechanical stability must fit the defect; surface chemistry affects protein adsorption and cell behavior; and local retention and release kinetics determine the duration of exposure to incorporated bioactive agents [19,20,21]. These parameters are particularly important in osteoporotic bone, where a transient osteogenic signal may be insufficient to overcome sustained oxidative, inflammatory, resorptive, and angiogenic dysfunction [22,23,24].

Material suitability is inherently defect dependent. A hydrogel may be appropriate for a small contained defect or as a delivery depot but cannot by itself provide the structural continuity needed for a load-bearing mandibular defect [25]. Conversely, a rigid 3D-printed scaffold may provide space maintenance and patient-specific geometry but still fail if pore architecture, vascularization, degradation, or biological signaling is inadequate. Modern tissue engineering therefore increasingly combines structural scaffolds with bioactive molecules, cells, extracellular vesicles, or responsive delivery systems rather than relying on passive osteoconduction alone [26,27].

## 5. Systemic and Bioactive Therapeutic Strategies

### 5.1. Melatonin

Melatonin was included because it targets several osteoporosis-associated barriers simultaneously [28,29,30]. Beyond its circadian function, melatonin has antioxidant and anti-inflammatory activity, can promote osteogenic differentiation, and can suppress osteoclastogenesis through receptor-dependent and receptor-independent mechanisms [31,32,33,34]. In osteogenic cells, melatonin has been associated with RUNX2, Osterix, ALP, collagen, osteocalcin, and osteopontin expression, while MT2-related signaling and modulation of RANKL/OPG and NF-κB pathways provide a mechanistic basis for effects on remodeling [35,36,37,38,39].

Importantly, the evidence is not limited to mechanistic studies. In ovariectomized rats with critical-size calvarial defects, Clafshenkel et al. reported greater regeneration with a calcium aluminate-melatonin scaffold than with the scaffold alone [40]. Dalla Costa et al. subsequently demonstrated that local melatonin increased mineral filling and newly formed bone in 5 mm calvarial defects in ovariectomized rats [41]. In a separate osteoporotic skeletal model, melatonin restored mitochondrial function of BMSCs and improved defect repair [42]. Most recently, Aydın and Çetiner reported enhanced histological new bone formation after systemic melatonin in grafted critical-size parietal defects in retinoic acid-induced osteoporosis, whereas vitamin E did not provide an additional benefit [43].

These studies constitute direct preclinical osteoporotic craniofacial evidence for melatonin, which is stronger than a purely mechanistic rationale. Nevertheless, direct clinical evidence in osteoporotic patients with craniomaxillofacial defects remains lacking. Local versus systemic administration, dose, carrier, release profile, and potential interaction with ongoing osteoporosis therapy remain unresolved. Melatonin should therefore be considered a preclinical regenerative candidate with unusually relevant craniofacial evidence, not an established clinical therapy for osteoporotic defects [44].

### 5.2. Vitamin D

Vitamin D was selected as a representative regulator of mineral homeostasis and bone-cell function. Correction of vitamin D deficiency is a standard component of osteoporosis management, but systemic skeletal benefit should not be conflated with defect regeneration [45,46,47,48]. Vitamin D receptors are expressed in osteoblast-lineage cells and participate in mineralization and remodeling, yet the regenerative effect of supplementation depends on baseline status, dose, route, and defect context [49,50,51,52].

Craniofacial preclinical data are mixed. In rat calvarial critical-size defects, oral vitamin D3 increased radiographic osteoid-area fraction, particularly when combined with guided bone regeneration [53]. A later study found that vitamins K2 and D3 enhanced guided bone regeneration in rat calvarial defects [54]. In contrast, Santinoni et al. found no significant increase in newly formed bone after systemic vitamin D in a 5 mm calvarial defect at an early 15-day endpoint, although qualitative signs of maturation were observed [55]. This heterogeneity illustrates why early marker or maturation effects should not automatically be described as regenerative efficacy.

For osteoporotic craniomaxillofacial regeneration specifically, direct evidence remains limited. Vitamin D is best interpreted as a clinically important host-status modifier and potential adjunct rather than a stand-alone regenerative therapy. Future studies should distinguish correction of deficiency from pharmacologic supraphysiologic dosing and should evaluate local delivery only when a plausible carrier and clinically relevant defect model are specified.

### 5.3. Vitamin E

Vitamin E was included as a contrasting antioxidant strategy. Tocopherols and tocotrienols can reduce lipid peroxidation and may influence inflammatory and osteogenic pathways, but their skeletal effects are heterogeneous and isoform dependent [56,57]. Much of the literature reports bone mineral density, trabecular architecture, biomechanical fracture outcomes, or cellular markers rather than direct craniofacial defect regeneration [58,59,60,61,62,63].

This distinction is reinforced by the 2026 osteoporotic cranial-defect study in which vitamin E, alone or in combination with melatonin, did not provide an additional regenerative advantage over grafting/melatonin conditions [43]. Thus, improved systemic antioxidant status or microarchitecture should not be treated as proof that vitamin E improves osteoporotic craniomaxillofacial defect healing. At present, vitamin E has lower direct evidence than melatonin and should be regarded as an adjunctive or mechanistically plausible strategy rather than a clinically prioritized regenerative intervention.

### 5.4. Teriparatide

Intermittent PTH(1–34), teriparatide, is an established anabolic osteoporosis therapy that stimulates osteoblast activity and survival and reduces sclerostin-mediated inhibition of Wnt signaling. Its clinical maturity distinguishes it from most agents discussed here [64,65]. However, systemic approval for osteoporosis and evidence for fracture outcomes do not by themselves establish efficacy for local osteoporotic craniomaxillofacial defects [66,67,68].

The strongest direct human oral-regeneration evidence comes from a randomized placebo-controlled trial by Bashutski et al., in which 40 patients undergoing periodontal surgery received teriparatide 20 micrograms/day or placebo for six weeks. At one year, radiographic resolution of periodontal osseous defects was greater with teriparatide, accompanied by improved clinical measures [69]. This is genuine human craniofacial regenerative evidence, but the population was not selected for osteoporosis. Preclinical craniomaxillofacial studies summarized in a recent systematic review further support anabolic effects in guided bone regeneration models [65].

Teriparatide therefore falls within the clinically investigated category for oral regeneration and has human oral-regeneration evidence, but the directness of evidence for osteoporotic craniomaxillofacial defects remains lower than its systemic osteoporosis credentials might imply. The principal translational questions are patient selection, timing relative to surgery, duration, cost, and interaction with prior or subsequent antiresorptive therapy rather than basic biological plausibility.

## 6. Locally Delivered Biological Strategies

### 6.1. Bone Morphogenetic Proteins

BMPs are potent osteoinductive members of the TGF-β superfamily. rhBMP-2 promotes osteogenic differentiation through receptor-mediated Smad signaling and downstream transcriptional programs including RUNX2 and Osterix. Unlike many experimental agents, rhBMP-2 has a well-developed clinical delivery pathway in craniofacial reconstruction; the clinically used construct combines rhBMP-2 with an absorbable collagen sponge (ACS), illustrating that efficacy is inseparable from carrier behavior [70,71,72]. In the United States, the INFUSE Bone Graft system, consisting of rhBMP-2 delivered on an absorbable collagen sponge, is approved for maxillary sinus augmentation and localized alveolar ridge augmentation associated with extraction sockets [70].

Human craniofacial evidence includes multiple defect types. In a multicenter randomized trial of 160 patients undergoing maxillary sinus-floor augmentation, rhBMP-2/ACS was compared with autogenous bone grafting and supported new bone formation and subsequent implant rehabilitation [73]. In an atrophic anterior maxilla trial, rhBMP-2/ACS with titanium-mesh space maintenance produced clinically relevant ridge augmentation comparable with autogenous grafting across most measurements [74]. A recent pilot randomized study also evaluated rhBMP-2-loaded ACS for alveolar ridge preservation after extraction [75]. These studies demonstrate direct human CMF regenerative evidence, but they are not osteoporosis-specific.

Delivery remains the principal limitation. ACS is biocompatible and clinically familiar but offers limited structural support and imperfect control of burst release and local retention. Calcium-phosphate carriers, composite scaffolds, hydrogels, and other controlled-delivery systems aim to improve spatial confinement and dose efficiency. Safety concerns include postoperative edema and unintended ectopic effects, while high dose and cost remain barriers [72,76]. BMP-2 is therefore clinically mature for selected craniofacial applications, yet its evidence should be described as human CMF evidence rather than direct evidence for osteoporotic CMF regeneration [76,77].

### 6.2. Platelet Concentrates: PRP and PRF

Platelet concentrates provide autologous fibrin matrices and endogenous mediators such as PDGF, TGF-β, VEGF, IGF, and other cytokines. PRF additionally functions as a provisional matrix that can support cell migration and a more sustained release profile than liquid PRP preparations [78]. These characteristics make platelet concentrates attractive local adjuncts, particularly where angiogenesis and early soft-tissue integration are relevant [78,79].

Osteoporosis-specific craniofacial evidence is limited but not absent. Durmuşlar et al. created bilateral 5 mm parietal critical-size defects in ovariectomized rats and compared untreated defects, injectable PRF, autogenous bone graft, and their combination. Histological and micro-CT findings showed enhanced regeneration in graft/PRF-treated groups, with the combined condition producing the highest mean new-bone volume ratio [80]. This constitutes direct osteoporotic cranial preclinical evidence. However, protocol sensitivity, donor-dependent variability, preparation method, and the difficulty of standardizing cellular and growth-factor content limit extrapolation.

PRF has broad clinical use in oral surgery, but clinical familiarity should not be mistaken for evidence of efficacy in osteoporotic defects. At present, PRF is best viewed as a locally delivered adjunct with biologically plausible angiogenic and wound-healing effects and emerging direct osteoporotic cranial evidence, rather than as a substitute for structural grafting in large defects [81,82,83,84].

## 7. Emerging Regenerative Strategies

### 7.1. Mesenchymal Stem Cell-Based Approaches

MSCs can contribute through osteogenic differentiation, paracrine signaling, immunomodulation, and angiogenic support. Clinical craniofacial translation is more advanced than a purely preclinical description would suggest. Kaigler et al. reported a randomized feasibility trial of bone-marrow-derived tissue repair cells for localized jawbone reconstruction [85], and contemporary systematic reviews identify a substantial body of prospective oral and craniofacial clinical studies [86,87,88,89,90].

However, osteoporosis introduces a major biological qualification: autologous MSCs from osteoporotic hosts may have reduced osteogenic potential, increased senescence, and altered responses to inflammatory and oxidative stress. Thus, the existence of human CMF stem-cell trials does not establish effectiveness in osteoporotic CMF defects. Future strategies may need cell preconditioning, biomaterial-mediated niche modulation, or cell-free products rather than simple transplantation.

### 7.2. Extracellular Vesicles and Exosome-Based Therapies

MSC-derived extracellular vesicles and exosomes can transport microRNAs, proteins, lipids, and regulatory cargo while avoiding some logistical and safety issues associated with viable-cell transplantation. Their principal limitations are rapid clearance, short local retention, heterogeneous cargo, isolation and characterization variability, and uncertain dose metrics [91].

Recent engineering studies illustrate how these limitations can be addressed. Xiang et al. incorporated engineered BMSC exosomes into a bone-targeting GelMA hydrogel, prolonging retention while reducing oxidative stress and promoting Wnt/β-catenin-related osteogenesis in 2.5 mm murine calvarial defects assessed at 4 and 8 weeks [92]. Anjum et al. developed a core–shell nanofiber system for spatiotemporal delivery of BMSC-derived exosomes and alendronate in an osteoporotic cranial-defect model, aiming to coordinate osteoblast and osteoclast activity; bone repair was assessed at 4 and 10 weeks [93]. These studies illustrate controlled local delivery, but they remain preclinical and do not establish clinical efficacy.

### 7.3. Hydrogels and Stimuli-Responsive Smart Biomaterials

Hydrogels should not be evaluated merely as containers for bioactive molecules. Matrix composition, cross-linking density, viscoelasticity, porosity, swelling, degradation, and adhesive behavior determine cell infiltration, vascularization, retention, and release kinetics. In osteoporotic defects, these features can be engineered to deliver prolonged or sequential signals that address oxidative stress, inflammation, impaired angiogenesis, and deficient osteogenesis [94,95,96].

Wang et al. recently reported a microenvironment-engineered hydrogel incorporating metformin and MgO nanoparticles that combined controlled release with antioxidant, anti-inflammatory, osteogenic, and angiogenic activity in osteoporotic bone-defect models [97]. Smart or stimuli-responsive systems extend this principle by linking release or material behavior to pH, ROS, enzymes, mechanical stimulation, or other pathological cues. A 2026 framework for oral and craniofacial regeneration emphasizes regulatory-network-oriented design rather than passive delivery [96].

The translational cost of this sophistication must be considered. Increasing material complexity can impair batch reproducibility, sterilization, scalability, quality control, storage, and regulatory approval. A smart material should therefore demonstrate a meaningful advantage over a simpler carrier, not merely additional mechanistic features.

### 7.4. Nanomaterials and Controlled Delivery Systems

Nanomaterials can improve local retention, protect bioactive cargo, and enable sustained or microenvironment-responsive delivery. Their relevance to osteoporotic bone regeneration is greatest when these functions are directed toward specific pathological barriers, including oxidative stress, inflammatory dysregulation, impaired osteogenesis, and excessive bone resorption. Cai et al. developed an injectable composite system incorporating mesoporous silica nanoparticles for the coordinated delivery of resveratrol and Mg^2+^. In osteoporotic rat femoral defects, the system modulated oxidative and inflammatory conditions while promoting angiogenic and osteogenic responses and enhancing defect-level bone regeneration [98].

More recently, Wang et al. developed a multifunctional hydrogel scaffold incorporating MgMn-layered double hydroxide nanosheets and calcium alendronate nanowires. The system was designed to reduce oxidative and inflammatory stress while simultaneously modulating osteoblast and osteoclast activity, and it accelerated bone-defect repair in osteoporotic rats [99]. These findings provide direct bone-regenerative support for nanomaterial-based strategies; however, both studies were performed outside the craniomaxillofacial region. Their evidence should therefore be regarded as osteoporotic skeletal rather than direct osteoporotic craniomaxillofacial evidence. Moreover, the technological capabilities of nanomaterials—including controlled release, local retention, and microenvironment-responsive activity—should not themselves be interpreted as evidence of regenerative efficacy. Translation will additionally require evaluation of degradation or clearance, off-target distribution, inflammatory responses, sterilization, manufacturing reproducibility, and performance in anatomically relevant craniomaxillofacial defects.

### 7.5. 3D-Printed and Bioprinted Scaffolds

3D printing provides control over macrogeometry, pore architecture, interconnectivity, and patient-specific fit. These advantages are particularly relevant to irregular craniofacial defects, but architectural fidelity alone does not overcome osteoporosis-associated biological impairment. Vascular invasion, mechanical stability, degradation, interface integration, and bioactive functionalization remain essential.

Yan et al. developed a peptide-functionalized 3D-printed PCL scaffold that recruited BMSCs and promoted vascular differentiation of endothelial progenitor cells, thereby improving vascularization and bone metabolic homeostasis in osteoporotic femoral-condylar defect models [100]. This study exemplifies a move from static structural scaffolds toward biologically instructive constructs. Bioprinting adds cells and bioactive matrices but introduces further challenges in cell viability, vascularization, mechanical fidelity, manufacturing, storage, and regulation.

## 8. Comparative Evidence and Translational Maturity

The strength and directness of evidence vary substantially across strategies. Melatonin and injectable PRF have direct preclinical osteoporotic cranial defect evidence [41,43,80]. Teriparatide and rhBMP-2 have stronger human craniofacial evidence and greater clinical maturity, but their key human trials were not specifically performed in osteoporotic CMF populations [69,73,74]. Vitamin D has supportive craniofacial preclinical evidence but inconsistent defect-level effects, while vitamin E remains supported largely by indirect skeletal and mechanistic findings [53,54,55,57].

Emerging approaches are mechanistically attractive because they can simultaneously modulate osteogenesis, inflammation, oxidative stress, angiogenesis, and delivery. However, most osteoporosis-specific hydrogel, exosome, nanomaterial, and 3D-printing studies remain preclinical. The anatomical relevance is heterogeneous: some recent studies use direct osteoporotic cranial-defect models, whereas others rely on osteoporotic long-bone defects or non-osteoporotic craniofacial models [93,97,98,100]. Their biological sophistication should therefore be separated from clinical readiness.

A two-dimensional framework avoids a misleading single ranking. A strategy may have an established or clinically investigated pathway in another indication but limited directness to osteoporotic CMF regeneration, as with teriparatide or rhBMP-2; alternatively, a strategy may have direct osteoporotic cranial preclinical evidence but remain preclinical for translation, as with melatonin. Table 1 summarizes representative studies using this framework, while the overall relationship between osteoporosis-associated barriers, therapeutic targets, interventions, delivery strategies, evidence directness, and translational maturity is illustrated in Figure 1.

Evidence directness should not be interpreted as evidence quality. A study may directly model an osteoporotic cranial defect yet remain limited by small sample size, short follow-up, a single animal model, absence of an active comparator, or reliance on surrogate rather than clinically meaningful endpoints. Conversely, a methodologically stronger clinical study conducted in a non-osteoporotic population may provide high-quality evidence for a specific intervention while remaining indirect with respect to osteoporotic defect regeneration. Accordingly, the present synthesis interprets evidence directness and translational maturity as two distinct descriptive dimensions, while individual study limitations are reported separately and should not be interpreted as a formal assessment of methodological quality or risk of bias.

**Table 1:** Representative Studies According to Evidence Directness and Translational Maturity.

Study	Model/OP	Site and Defect	Intervention/Delivery	Follow-Up	Endpoint	Main finding	Evidence	Limitation	Translational Maturity
Clafshenkel 2012 [40]	OVX rat	Calvarial critical-size	Calcium-aluminate + melatonin; local scaffold	3/6 mo	Fluorochrome; new bone	Greatest fluorochrome-labeled bone regeneration with CA-Mel scaffold at 3 and 6 mo.	OP cranial; direct preclinical	Older model; carrier-specific	Preclinical
Dalla Costa 2024 [41]	OVX rat	Bilateral 5 mm calvarial	Melatonin ± Bio-Oss; local powder/xenograft	45 d	micro-CT; histomorph.	Melatonin significantly increased mineral filling and newly formed bone in OVX defects (*p* < 0.05).	OP cranial; direct preclinical	Animal model; short follow-up	Preclinical
Aydın 2026 [43]	Retinoic-acid OP rat	8 mm parietal critical-size + graft	Melatonin 10 mg/kg/day; vitamin E 20 mg/kg/day; systemic i.p.	28 d	Histology: new bone; osteoblasts/osteoclasts; fibrosis	Pronounced new-bone formation with melatonin; vitamin E provided no additional benefit.	OP cranial; direct preclinical	Single model; semiquant. histology	Preclinical
Varlıhan 2026 [54]	Healthy rat	Bilateral 5 mm calvarial critical-size + GBR	Vitamin D3 and/or K2; systemic oral	8 wk	Histomorph.; OCN, RUNX2, IL-1β	Vitamin groups increased new bone; combined K2 + D3 showed the greatest regeneration in GBR defects.	CMF non-OP; preclinical	Not osteoporosis-specific	Preclinical
Bashutski 2010 [69]	Human periodontitis	Periodontal osseous defects	Teriparatide 20 µg/day; systemic injection	1 y	Radiographic defect resolution; clinical	1-y radiographic defect resolution: 29% with teriparatide vs. 3% with placebo (*p* < 0.001).	CMF non-OP; human	Small trial; periodontal indication	Clinically investigated
Triplett 2009 [73]	Human	Maxillary sinus augmentation	rhBMP-2 1.5 mg/mL; ACS carrier	6 mo + 2 y	CT; histology; implant outcomes	6-mo bone-height gain: 7.83 ± 3.52 mm (rhBMP-2/ACS) vs. 9.46 ± 4.11 mm (autograft).	CMF non-OP; human	Not OP-specific; dose/carrier	Established clinical use
de Freitas 2013 [74]	Human	Atrophic anterior maxilla	rhBMP-2/ACS vs autograft; ACS + Ti mesh	6 mo	Ridge gain; implant outcomes	6-mo horizontal gain: 3.2 ± 0.9 mm (rhBMP-2/ACS) vs. 3.7 ± 1.4 mm (autograft; *p* = 0.31).	CMF non-OP; human	Small clinical trial; non-OP	Clinically investigated
Durmuşlar 2024 [80]	OVX rat	Bilateral 5 mm parietal	I-PRF ± autograft; local clot/graft	4 wk	micro-CT; histology	I-PRF + autograft produced the highest mean new-bone volume ratio.	OP cranial; direct preclinical	Small animal study; PRF variability	Preclinical
Kaigler 2013 [85]	Human	Localized jaw defects	Bone-marrow tissue repair cells; cell transplant	6/12 wk	Clinical; radiographic	6-wk radiographic bone fill: 78.9% (TRC) vs. 55.3% (GBR; *p* = 0.01).	CMF non-OP; human	Early-phase study; non-OP	Clinically investigated
Xiang 2025 [92]	Healthy C57BL/6 mouse	2.5 mm full-thickness calvarial defect	Engineered BMSC exosomes; bone-targeted GelMA hydrogel	4/8 wk	micro-CT; histology; oxidative/osteogenic endpoints	Improved local retention and calvarial bone repair at 4 and 8 wk.	CMF non-OP; preclinical	Not osteoporosis-specific	Preclinical
Anjum 2026 [93]	Osteoporotic rat	Cranial bone defect	BMSC exosomes + alendronate; core–shell nanofiber scaffold	4/10 wk	micro-CT; histology; osteoblast/osteoclast activity	Dual exosome/alendronate delivery enhanced bone repair at 4 and 10 wk.	OP cranial; direct preclinical	Complex manufacture; no human evidence	Preclinical
Cai 2025 [98]	Osteoporotic rat	Femoral bone defect	Resveratrol + Mg^2+^ nanoparticles; injectable composite hydrogel	6/12 wk	micro-CT; histology; ROS/immune/angiogenic endpoints	Accelerated osteoporotic femoral-defect regeneration with coordinated antioxidant, immunomodulatory, angiogenic, and osteogenic effects.	OP skeletal non-CMF; indirect	Non-CMF; nanoparticle safety/scale	Preclinical

Note: Evidence directness describes relevance to osteoporotic craniomaxillofacial defect regeneration and should not be interpreted as a measure of methodological quality. Cranial-defect evidence is distinguished from dentoalveolar, periodontal, peri-implant, sinus, and mandibular evidence where applicable.

## 9. Clinical and Translational Considerations

Systemic host-modulating therapies and locally delivered regenerative interventions are fundamentally different paradigms. Systemic therapies affect the entire skeleton and are constrained by systemic exposure, indication, duration, adverse effects, and interactions with other osteoporosis drugs. Local therapies depend on carrier selection, concentration, retention, release kinetics, surgical handling, degradation, and defect integration. Evidence for systemic skeletal efficacy should therefore not be extrapolated directly to local regenerative efficacy or vice versa.

Clinical reality also requires consideration of ongoing antiresorptive or anabolic therapy. Patients presenting for implants, augmentation, or reconstructive procedures may already be receiving bisphosphonates, denosumab, teriparatide, or other osteoporosis treatments. Contemporary consensus evidence suggests that antiresorptive therapy for osteoporosis does not necessarily increase implant failure and generally does not require routine discontinuation before implant placement, although MRONJ risk and treatment history remain relevant [101,102]. Existing medication, dose, duration, and timing relative to surgery should therefore be reported as potential modifiers of regenerative outcomes.

Finally, defect type must guide interpretation. A calvarial critical-size defect is useful for standardized preclinical screening but does not reproduce the loading, dental contamination risk, marrow environment, or functional requirements of a mandibular or peri-implant defect. Likewise, sinus augmentation, ridge augmentation, extraction sockets, and segmental defects require different structural and biological solutions. Translation should therefore proceed from a clinically matched model rather than from the assumption that all craniofacial bone behaves as a single regenerative site.

## 10. Translational Gaps and Future Research Priorities

The principal gap is not simply a shortage of studies but a shortage of comparable studies that answer clinically relevant questions. Future preclinical work should select osteoporosis induction according to the target patient population and verify that the model produces a meaningful regenerative impairment. Ovariectomy, aging, glucocorticoid exposure, and other models reproduce different biological components and should not be treated as equivalent. Species, defect dimensions, criticality, anatomical site, and follow-up must be reported and considered when comparing efficacy.

Priorities should differ according to the main limitation of each strategy. Interventions with reproducible defect-level evidence and acceptable safety/delivery profiles should move toward carefully selected clinical testing. Strategies with strong biology but poor retention should first undergo delivery optimization. Approaches supported predominantly by cellular markers, BMD, or non-craniofacial outcomes need direct osteoporotic CMF proof-of-concept before clinical translation. Complex smart materials should demonstrate an advantage over simpler carriers sufficient to justify manufacturing and regulatory burden.

A standardized core outcome set would improve comparability. Direct regenerative endpoints should include micro-CT measures of defect fill and BV/TV, histomorphometric new-bone area and residual material, evidence of defect bridging or structural integration, and appropriately timed assessment of mature remodeling. Vascular endpoints should be included when angiogenesis is a therapeutic target; mechanical or functional testing is essential for load-bearing defects; implant studies should report relevant osseointegration measures. Baseline osteoporosis phenotype and concomitant therapy should be documented. BMD, osteogenic markers, or systemic microarchitecture should remain supportive outcomes rather than substitutes for defect regeneration.

Rather than defining one universally optimal model, future studies should match the osteoporosis model and defect to the intended indication. A clinically useful model should combine a verified osteoporotic phenotype with an anatomically relevant CMF defect in which impaired regeneration is measurable, use a follow-up long enough to assess mature repair, and include outcomes that can be translated to the proposed clinical procedure.

## 11. Limitations of the Review

This review has several limitations. First, it was designed as a narrative rather than a systematic review; consequently, literature identification and study selection were relevance-based and were not conducted using a formal systematic-review screening protocol. Second, the literature search was limited to PubMed and Web of Science, and relevant studies indexed exclusively in other databases may therefore have been missed. Third, the purposive selection of representative studies for detailed comparison in Table 1 introduces a potential risk of selection bias, although explicit relevance criteria were applied to improve transparency. Fourth, no formal risk-of-bias or study-quality assessment tool was applied. Evidence directness should therefore not be interpreted as a surrogate for methodological quality, and comparisons among interventions should be considered descriptive rather than formal comparative efficacy rankings. Fifth, the categories of evidence directness and translational maturity constitute an author-defined descriptive framework with operational definitions provided in Section 2 rather than a validated evidence-grading system. Finally, substantial heterogeneity in osteoporosis induction, species, anatomical site, defect geometry and criticality, intervention dose and delivery, follow-up duration, and regenerative endpoints limits direct comparison across studies. These limitations should be considered when interpreting the translational conclusions of this review.

## 12. Conclusions

Osteoporotic craniomaxillofacial bone regeneration remains supported by a limited and heterogeneous evidence base. Although several systemic agents, biologics, cell-derived products, and engineered delivery systems have demonstrated regenerative effects, most evidence derives from preclinical studies, non-craniomaxillofacial osteoporotic models, or craniomaxillofacial models not specifically designed to reproduce osteoporosis. Direct evidence from osteoporotic craniomaxillofacial defects is sparse and is dominated by small animal studies. Accordingly, the presence of anatomically relevant preclinical findings should be interpreted as proof of concept rather than as an established evidence base or evidence of clinical translational readiness.

Current findings nevertheless identify several biologically relevant therapeutic targets, including impaired osteogenesis, excessive bone resorption, oxidative and inflammatory stress, and compromised angiogenic support. Future studies should determine whether interventions directed toward these barriers provide reproducible regenerative benefit in anatomically and clinically relevant osteoporotic craniomaxillofacial models, particularly at alveolar, periodontal, peri-implant, and mandibular sites. Standardized defect models, clinically meaningful regenerative endpoints, adequate controls, longer follow-up, and direct comparisons between treatment strategies will be necessary before relative efficacy can be established. Ultimately, well-designed clinical studies in patients with osteoporosis are required before these approaches can be considered evidence-based strategies for routine craniomaxillofacial regeneration.

## Figures and Tables

**Figure 1:**
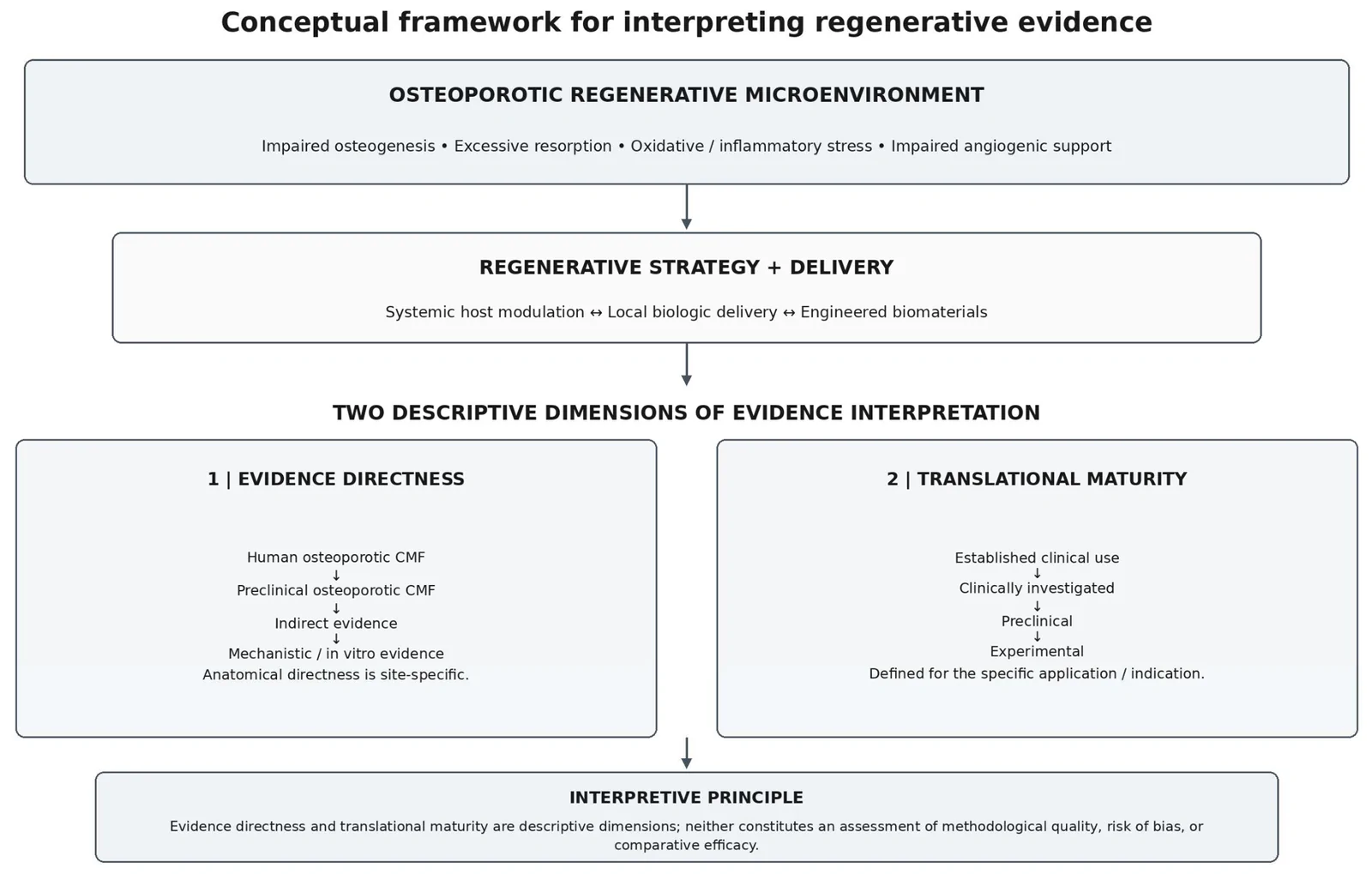
Conceptual framework for interpreting regenerative evidence in osteoporotic craniomaxillofacial bone defects. Regenerative strategies are considered in relation to the pathological barriers they target and their delivery approach, while the resulting evidence is descriptively interpreted across two dimensions: evidence directness and translational maturity. Evidence directness reflects the relationship of the study population/model, anatomical site, and regenerative endpoint to the review question, whereas translational maturity reflects the stage of application according to the operational categories defined in Section 2. These dimensions are descriptive and should not be interpreted as assessments of methodological quality, risk of bias, or comparative efficacy. Anatomical directness remains site-specific; therefore, evidence from cranial defects should not be considered equivalent to evidence from alveolar, periodontal, peri-implant, sinus, or mandibular defects.

## Data Availability

No new data were created or analyzed in this study. Data sharing is not applicable to this article.

## Abbreviations

The following abbreviations are used in this manuscript:

ACS	Absorbable collagen sponge
ALP	Alkaline phosphatase
BMD	Bone mineral density
BMP/BMPs	Bone morphogenetic protein(s)
BMSC/BMSCs	Bone marrow mesenchymal stem cell(s)
BV/TV	Bone volume/total volume
CMF	Craniomaxillofacial
GBR	Guided bone regeneration
GelMA	Gelatin methacryloyl
I-PRF	Injectable platelet-rich fibrin
IGF	Insulin-like growth factor
MRONJ	Medication-related osteonecrosis of the jaw
MSC/MSCs	Mesenchymal stem cell(s)
NF-κB	Nuclear factor kappa B
OPG	Osteoprotegerin
OVX	Ovariectomized/ovariectomy
PCL	Polycaprolactone
PDGF	Platelet-derived growth factor
PRF	Platelet-rich fibrin
PRP	Platelet-rich plasma
PTH	Parathyroid hormone
RANK	Receptor activator of nuclear factor-κB
RANKL	Receptor activator of nuclear factor-κB ligand
rhBMP-2	Recombinant human bone morphogenetic protein-2
ROS	Reactive oxygen species
RUNX2	Runt-related transcription factor 2
TGF-β	Transforming growth factor-β
VEGF	Vascular endothelial growth factor.
